# The Impact of Patient Characteristics and Tumor Biology on the Accuracy of Preoperative Staging of Colon Cancer in Denmark. A Nationwide Cohort Study

**DOI:** 10.3390/cancers13174384

**Published:** 2021-08-30

**Authors:** Malene Roland V. Pedersen, Søren Rafael Rafaelsen, Jan Lindebjerg, Torben Frøstrup Hansen, Hans Bjarke Rahr

**Affiliations:** 1 Department of Radiology, Vejle Hospital, University Hospital of Southern Denmark, DK-7100 Vejle, Denmark; Soeren.Rafael.Rafaelsen@rsyd.dk; 2 Department of Regional Health Research, University of Southern Denmark, DK-7100 Vejle, Denmark; jan.lindebjerg@rsyd.dk (J.L.); Torben.Hansen@rsyd.dk (T.F.H.); hans.rahr@rsyd.dk (H.B.R.); 3 Danish Colorectal Cancer Center South, Vejle Hospital, University Hospital of Southern Denmark, DK-7100 Vejle, Denmark; 4 Department of Pathology, Vejle Hospital, University Hospital of Southern Denmark, DK-7100 Vejle, Denmark; 5 Department of Oncology, Vejle Hospital, University Hospital of Southern Denmark, DK-7100 Vejle, Denmark; 6 Department of Surgery, Vejle Hospital, University Hospital of Southern Denmark, DK-7100 Vejle, Denmark

**Keywords:** mismatch repair, colon cancer, registry study, tumor staging

## Abstract

**Simple Summary:**

The roles of mismatch repair and other colon tumor characteristics were investigated in a nationwide registry study with data extracted from the Danish Colorectal Cancer Group. Mismatch repair can arise during DNA replication. In this study, 6102 patients were included with a median age of 72 (range 23–97 years). The mismatch repair was deficient in 24% and proficient in 76%. Mismatch repair deficiency impacted the accuracy of the preoperative staging of colon cancer. In the future, mismatch repair status should be taken into consideration in the clinical staging of colon cancer.

**Abstract:**

Background: Colon cancer is a common disease in western populations. The aim of this study was to assess the impact of mismatch repair (MMR) deficiency and other patient and tumor characteristics on the accuracy of preoperative staging by comparing histopathological T- and N-categories of the resected specimen with the preoperative clinical stage in a nationwide cohort of patients treated for colon cancer by elective bowel resection with curative intent. Methods: A register study of a cohort extracted from the Danish Colorectal Cancer Group (DCCG) database, which holds prospective data on all new cases of colon and rectum cancer in Denmark. Patients diagnosed with colon cancer and treated with an elective bowel resection with curative intent in the years 2016–2019 were analyzed. Results: A total of 6102 patients were included (*n* = 3161 (52%) men and *n* = 2941 (48%) women) with a median age of 72 years (range 23–97 years). MMR was deficient in 24% of the patients and proficient in 76%. MMR deficiency, tumor sidedness and histopathological type were significant predictors of the accuracy of preoperative staging of colon cancer in univariate and multivariate analysis. MMR status in particular showed a strong impact on the risk of overstaging. Conclusions: MMR deficiency, but also tumor sidedness and to some degree histopathological type, impacted the accuracy of preoperative staging of colon cancer. MMR status should be taken into consideration in everyday clinical staging.

## 1. Introduction

Colon cancer is a common disease in the Western world [1,2]. Most patients are treated by surgery, but very often in combination with pre- and/or post-operative chemotherapy and biological treatment. Treatment regimens are becoming increasingly complex, and accurate staging before treatment is crucial. Previous studies have shown limited accuracy of the standard diagnostic workup with contrast-enhanced computed tomography (CT) scans, particularly with regard to involvement of regional lymph nodes (N-category), which is often overestimated [3,4,5,6]. Recently, Erbs and colleagues found overstaging with regard to the N-category to be more common in patients whose colon tumors were mismatch repair (MMR) deficient (dMMR) compared with patients with MMR proficient (pMMR) tumors [7]. The study was a single-center series of patients retrospectively reviewed by an expert radiologist; however, it seems warranted to elucidate the impact of MMR status on routine clinical practice by conducting a larger, population-based study. Other patient and tumor characteristics, such as age, sex, body mass index (BMI) and tumor location (left vs. right colon), may also influence the accuracy of preoperative staging. Two recent Swedish studies were not able to demonstrate this unequivocally, but this may be because only Kappa statistics were used [8,9]. Since MMR status may be associated with both sidedness and histopathology, we suggest that adjusted, separate multivariate analysis of under- and over-staging may provide new and more useful information. Other plausible predictors for correct staging, e.g., histopathological type and discussion at a multidisciplinary team conference (MDT), should be included as potential covariates. A detailed understanding of CT performance is an essential basis for both further technical improvements and the current considerations on replacing CT as the standard modality with other imaging modalities that seem to perform better in some respects [10,11,12,13,14,15,16].

The aim of this study was to assess the impact of MMR deficiency and other patient and tumor characteristics on the accuracy of preoperative T and N staging by comparing the preoperative T- and N-categories (cTNM) with the histopathological T- and N-categories of the resected specimen (pTNM) in a nationwide cohort of patients treated for colon cancer by elective bowel resection with curative intent.

## 2. Materials and Methods

The study was approved locally by the local National Data Protection Agency. According to national law, the current study did not require patient consent or approval by the National Health Research Ethics Committee since there was no biomedical intervention. Permission to use the data was granted by the national quality assurance authority (Regionernes Kliniske Kvalitetsudviklingsprogram, RKKP) (No. DCCG-2020-10-14).

### 2.1. Design and Data Sources

This was a register-based study of a patient cohort extracted from the Danish Colorectal Cancer Group (DCCG) database [17]. All new cases of colon and rectum cancer in Denmark are entered prospectively into this nationwide database founded by the DCCG and hosted by the RKKP. The database holds virtually all (>98%) patients diagnosed with colon cancers in Denmark since May 2001. The database has a high completeness of data on patient characteristics, diagnostic workup, treatment, short-term outcomes and histopathology. Since 2016, all patients have been assigned a clinical disease stage (cTNM) based on the diagnostic investigations before treatment and the assessment and discussion in the MDT.

### 2.2. Data Collection

We extracted 9167 patients classified by the surgeons as having primary colon cancer, diagnosed during the calendar years 2016–2019 and treated with an elective operative procedure with curative intent. Patients were excluded if the operative procedure did not include formal bowel resection and thus did not produce a resection specimen for histopathological examination (*n* = 499). We also excluded patients in whom the current definitive procedure had been preceded by a previous emergent procedure (*n* = 424) or local resection of a malignant polyp (*n* = 739), i.e., in whom the quality of preoperative imaging might not be optimal. Patients in whom the information on synchronous tumors was either missing or affirmative (*n* = 429), or whose tumor was recorded by the pathologist as situated in the rectum (*n* = 67), were also excluded.

To ascertain the location of the tumor in question, we conducted the following validation procedure: Tumor locations were classified as right-sided or left-sided, defining midgut tumors (i.e., caecum, ascending colon, right colonic flexure and transverse colon) as right-sided and hindgut tumors (i.e., left flexure, descending colon, and sigmoid colon) as left-sided. Tumors were subsequently allocated to either the right-sided or left-sided group if the tumor location recorded by the surgeon and the pathologist agreed with regard to side (*n* = 6830), or if the surgeon had recorded the tumor location as “unspecified”, but the pathologist had recorded a specific location compatible with the operative procedure recorded (*n* = 8). Tumors were defined as transitional if regarded by the surgeon as situated in the transverse colon, and by the pathologist as situated in the left flexure, or vice versa (*n* = 60). The remaining 111 patients were excluded owing to missing or doubtful tumor location.

Finally, we excluded patients in whom information on neoadjuvant treatment (*n* = 277), or distant metastasis at the time of diagnosis (*n* = 519), was either missing or affirmative. Thus, 6102 patients remained for analysis: 3610 right-sided and 2441 left-sided, whereas 51 had a tumor on the midgut/hindgut transition.

### 2.3. Diagnostic Tests

National guidelines are in place for diagnosis and treatment of colorectal cancer in Denmark. A thoracoabdominal CT is the standard imaging modality for staging in colon cancer before treatment, although sometimes it is deemed necessary to add other imaging techniques, such as ultrasonography or positron emission tomography (PET).

The imaging techniques used for preoperative staging used to be recorded in the DCCG database, but this practice was discontinued in the summer of 2019, leaving missing values in 1133 patients (19%) of the present cohort. Since an abdominal CT was recorded in >97% of patients from the years 2016–2018, we assume that CT scans have been performed in nearly all patients. No details on the technical aspects of the imaging used are available in the DCCG database, and the data registered before 2019 show that modalities other than CT have been applied in 2–3% of patients. It is important, therefore, to understand that the present study is an assessment of the overall diagnostic performance of the MDT rather than of any particular imaging modality. The gold standard (histopathological examination) and MMR analyses were also assumed to be conducted according to national guidelines. MMR deficiency (dMMR) was regarded as present if any of the individual markers (MLH1, MSH2, MSH6 and PMS2) were deficient.

### 2.4. Data Analysis

We used descriptive methods to summarize baseline data. Overall measures of accuracy (sensitivity, specificity and positive predictive value (PPV), negative predictive value (NPV), false positive rate (FPR), false negative rate (FNR), false discovery rate (FDR) and false omission rate (FOR)) were estimated with exact 95% confidence intervals (CI). We focused on the ability to detect early vs. advanced tumor stage and aggregated T-categories into T0–2 vs. T3–4 and N-categories into N0 vs. N+.

We selected FPR and FNR as our main parameters of interest, since they imply a direct comparison of the diagnostic method with the gold standard and are independent of disease prevalence. They were calculated separately for the T-category (T-FPR, T-FNR) and the N-category (N-FPR, N-FNR). FPR and FNR were cross-tabulated against MMR status and other plausible predictors for inaccurate staging (age, sex, BMI, comorbidity as measured by the Charlson score (CCI), histopathology, tumor location (right vs. left) and discussion at an MDT meeting) and subsequently regressed on selected predictors in univariate and multivariate logistic regression analyses.

FDR (=1-PPV) and FOR (=1-NPV) may be more readily useful to the clinician in decision making and patient counseling because they provide a direct answer to the clinical question: “What is the chance that my assessment is wrong?”. These statistics were cross-tabulated against significant predictors.

### 2.5. Statistical Analysis

All analyses and data management were performed using STATA Statistical Software (version 16, STATA Corporation, 4905 Lakeway Drive, College Station, TX, USA). Final conclusions were based on a significance level of 0.01, although higher levels (0.2, 0.05) were used for exploration and variable selection. Confidence intervals (CI) are 95%.

## 3. Results

### 3.1. Patients and Tumor Characteristics

A total of 6102 patients were analyzed (*n* = 3161 (52%) men and *n* = 2941 (48%) women). Patient, tumor and treatment characteristics are described in Table 1. Table 2 shows overall measures of accuracy.

### 3.2. FPR, FNR, FDR and FOR

Table 3 shows unadjusted logistic regression of FPR and FNR. In the unadjusted logistic regression, T-FPR seemed to increase slightly with age (*p* < 0.2), albeit with an odds ratio (OR) very close to 1. T-FNR increased with male sex and comorbidity (both as a dichotomous and continuous variable) at the *p* < 0.2 level and decreased significantly with histopathological poor differentiation and dMMR status at the *p* < 0.001 level. N-FPR decreased with male sex, discussion at an MDT meeting (*p* < 0.2) and comorbidity (*p* < 0.001) and increased significantly with dMMR status, right-sided tumors and histopathology other than adenocarcinoma not otherwise specified (NOS) (*p* < 0.001). N-FNR seemed to increase somewhat with comorbidity and an MDT meeting (*p* < 0.2) and decrease significantly with right-sided tumors, dMMR status and histopathology other than adenocarcinoma NOS (*p* < 0.001).

Before proceeding with adjusted analyses, the importance of sidedness and MMR status was further explored by cross-tabulations. Significant associations were found between dMMR status and sex (66% females), sidedness (92% right-sided tumors) and histopathology (45% other than adenocarcinoma NOS vs. 11% in the pMMR group). Similarly, nodal involvement was less common among dMMR patients (27% vs. 38% in pMMR patients) and somewhat more common in right-sided than in left-sided pMMR tumors (40% vs. 36%). Right-sided tumors were also more often locally advanced (T3–4) (75%) than left-sided tumors (67%).

For the adjusted analyses, we selected sidedness, dMMR status and histopathology as our predictors of interest, and age, sex, BMI and MDT meeting as adjusting factors. Comorbidity as measured by the CCI score was included among the potential confounders, since CCI is not an exact measure of all present comorbidities and may not be as unequivocal and simple as the predictors of interest.

Baseline logistic regression models with T-FPR, T-FNR, N-FPR and N-FNR as dependent variables and the abovementioned potential confounders as covariates were constructed, and the predictors of interest were entered one at a time and in combination to provide adjusted analyses.

In the adjusted analyses, none of the predictors of interest showed a significant association with T-FPR.

T-FNR was significantly decreased with low tumor differentiation (with OR estimates ranging from 0.47 to 0.52) and dMMR (OR, 0.64–0.72), but not with sidedness. N-FPR was significantly increased by right-sidedness (OR, 1.48–1.73), dMMR (OR, 1.41–1.74) and low tumor differentiation (OR, 1.62–1.87), although histopathology became statistically insignificant when the other predictors were introduced. N-FNR was significantly decreased with right-sidedness (OR, 0.44), dMMR (OR, 0.59) and low tumor differentiation (OR, 0.46–0.57), although only right-sidedness remained significant (OR, 0.48–0.49) when the two other predictors were introduced.

Empirical data for FPR, FNR, FDR and FOR are tabulated against the two persistent predictors (sidedness and MMR status) in Table 4. The *n* values in the table are the denominators of the statistic in question and reflect the varying size of the subgroups. Some are rather small (e.g., left-sided dMMR tumors), and this explains the varying width of the confidence intervals. Incorrect preoperative staging was common, particularly in terms of T understaging and N overstaging. The latter was pronounced for dMMR patients.

## 4. Discussion

The aim of this study was to assess the impact of MMR deficiency and other patient and tumor characteristics on the accuracy of preoperative T and N staging in colon cancer. We found a significant and persistent association between dMMR and overestimation of the N-category, but we also found a significant impact of histopathology and tumor sidedness. Reactive lymph node enlargement in dMMR cancers is to be expected, because inability to repair mismatch results in accumulation of mutations, leading to the production of immune-response-eliciting proteins [18,19]. Additionally, there is a growing interest in regarding right- and left-sided cancers as distinct biological and clinical entities [20,21,22,23,24,25,26,27,28,29].

FPR and FNR were chosen as dependent variables since they imply direct comparisons of the diagnostic method and the gold standard and are independent of disease prevalence (in casu T3–4 and N+). However, these traditional measures of accuracy are often perceived by the average clinician as rather indirect indicators of the reliability of the diagnostic method. To translate our findings into figures that are easy to understand in a clinical context, we also calculated FDR and FOR, which are shown in Table 4. FDR and FOR may be regarded as the percentage of clinical T- and N-categories that ultimately turn out to be wrong. It may be noted that the importance of sidedness for N staging seems to fade or even reverse when results are presented as FDR and FOR, whereas the differences between dMMR and pMMR tumors become more pronounced. This may be explained by the dependence of FDR and FOR on prevalence. For example, increasing prevalence (e.g., of N+ status) tends to attenuate the effect of FPR on FDR, whereas decreasing prevalence tends to enhance it. The data shown in Table 4 are consistent with this explanation, when the differences in T- and N-categories (i.e., the prevalence of T3–4 and N+) between right- and left-sided tumors, and dMMR and pMMR tumors, are taken into consideration. This observation exemplifies the complex interplay of the determinants of test performance and emphasizes the importance of the clinical setting on its real-life effectiveness.

In this study, we found a sensitivity of 68% and specificity of 78% for the advanced cT-category (Table 2). We have no information on the expertise of the radiologist, and in general, there is limited information available on the importance of the radiologist’s level of expertise for accurate staging. CT staging can be challenging due to the anatomic structures and bowel movement and requires experience. Hong et al. compared performance between radiologists receiving feedback and others receiving no feedback and found better performance after feedback [30]. On the other hand, a meta-analysis found that magnetic resonance imaging (MRI) can reliably distinguish T1–T2 from T3–T4 (sensitivity 96% and specificity 70%) [31], suggesting that MRI may have potential to replace CT as the standard modality for colon cancer in the future.

This study has some limitations. Formally, the study is retrospective, and for some variables, the number of missing data is not negligible. In addition, we have no information on the actual scanning dates, the skill level of the radiologists and any variation in techniques and protocols. A strength of the current study, on the other hand, is the very large, population-based dataset based on prospectively registered data from a national database. Further strengths of the current study are that routine data on MMR were available in most patients, and more generally, that all data were routinely collected and therefore as unbiased and representative for a real-life clinical setting as possible.

In summary, we showed with prevalence-independent statistics that MMR status in particular, but also other clinical and biological characteristics, may influence the accuracy of the preoperative staging. We also found, however, that in the everyday clinical setting, the importance of some of these factors may be further modified by the mere prevalence of the pathology in question. Table 4 presents empirical data from our study population in a manner that should be easy to understand and apply in a clinical setting.

## 5. Conclusions

In conclusion, MMR deficiency, but also tumor sidedness and to some degree histopathological type, impacts the accuracy of preoperative staging of colon cancer. For everyday clinical staging, MMR status should be taken into consideration.

## Figures and Tables

**Table 1:** Baseline characteristics of 6102 patients with colon cancer.

Characteristic	Quantity	Missing (%)
Age (median, range)	72 (23–97)	
Sex (*n*, %)		
Female	2941 (48%)	
Male	3161 (52%)	
BMI (kg/m^2^) (median, IQR)	26 (23–29)	118 (2%) *
Charlson score (CCI)		
0 (*n*, %)	2728 (45%)	
1	858 (14%)	
2	1526 (25%)	
3+	990 (16%)	
Tumor side		
Right (*n*, %)	3610 (59%)	
Left	2441 (40%)	
Transition	51 (1%)	
Preoperative clinical T-category (cT)		947 (16%)
cT0 (*n*, % of non-missing)	66 (1%)	
cT1	444 (9%)	
cT2	1760 (34%)	
cT3	2413 (47%)	
cT4	472 (9%)	
Preoperative clinical N-category (cN)		838 (14%)
cN0 (*n*, % of non-missing)	3067 (58%)	
cN1	1472 (28%)	
cN2	725 (14%)	
Histopathology		3 (0%)
Adenocarcinoma NOS (*n*, % of non-missing)	4899 (80%)	
Low differentiated carcinoma	394 (6%)	
Mucinous carcinoma	692 (11%)	
Other **	114 (2%)	
MMR status		199 (3%)
Deficient (dMMR) (*n*, % of non-missing)	1425 (24%)	
Proficient (pMMR)	4478 (76%)	

* Including 17 BMI values discarded on suspicion of registration errors (weight < 10 kg, height > 250 cm, BMI > 50). ** Signet-cell, undifferentiated, or medullary.

**Table 2:** Overall measures of accuracy, clinical vs. histopathological category in colon cancer.

Measure	Calculated	95% CI *	
	T-Category (T0–2 vs. T3–4)—All Patients		
Sensitivity	0.68	0.66	0.69
Specificity	0.78	0.76	0.80
PPV	0.90	0.89	0.91
NPV	0.46	0.44	0.48
FPR	0.22	0.20	0.24
FNR	0.32	0.31	0.34
FDR	0.10	0.09	0.11
FOR	0.54	0.52	0.56
	N-category (N0 vs. N+)—All Patients		
Sensitivity	0.55	0.53	0.57
Specificity	0.66	0.64	0.67
PPV	0.47	0.45	0.49
NPV	0.72	0.71	0.74
FPR	0.34	0.33	0.36
FNR	0.45	0.43	0.47
FDR	0.53	0.51	0.55
FOR	0.28	0.26	0.29

* Confidence interval.

**Table 3:** Unadjusted logistic regression analysis of accuracy on predictors.

Variable	OR *	*p*	95% CI **	
T-category—false positive rate				
Age	1.0179	0.014	1.0036	1.0324
Sex	1.0043	0.974	0.7758	1.3000
BMI	0.9877	0.371	0.9612	1.0149
Comorbidity (+/−)	0.9007	0.430	0.6948	1.1676
Comorbidity (cont)	0.9664	0.447	0.8848	1.0554
MDT meeting	0.8558	0.298	0.6385	1.1473
Side (left vs. right)	0.8546	0.239	0.6577	1.1103
Histopathology				
-Adenocarcinoma NOS	1 (reference)			
-Low differentiated	1.2222	0.541	0.6427	2.3244
-Mucinous	1.6667	0.554	0.7004	1.9432
-Other	3.6667	0.025	1.1726	11.4651
dMMR ***	1.3334	0.425	0.8833	1.5415
T-category—false negative rate				
Age	0.9958	0.208	0.9893	1.0023
Sex	1.1145	0.120	0.9721	1.2777
BMI	1.0090	0.205	0.9951	1.0231
Comorbidity (+/−)	1.1446	0.054	0.9975	1.3135
Comorbidity (cont)	1.0560	0.015	1.0105	1.1036
MDT meeting	1.0608	0.502	0.8929	1.2602
Side (left vs. right)	1.0338	0.646	0.8972	1.1912
Histopathology				
-Adenocarcinoma NOS	1 (reference)			
-Low differentiated	0.4717	<0.001	0.3492	0.6374
-Mucinous	1.0762	0.472	0.8812	1.3144
-Other	0.6848	0.138	0.4150	1.1300
dMMR ***	0.6582	<0.001	0.5569	0.7779
N-category—false positive rate				
Age	1.0025	0.492	0.9953	1.0098
Sex	0.8387	0.015	0.7276	0.9668
BMI	0.9955	0.554	0.9809	1.0104
Comorbidity (+/−)	0.7960	0.002	0.6902	0.9182
Comorbidity (cont)	0.9164	<0.001	0.8730	0.9618
MDT meeting	0.8546	0.068	0.7218	1.0120
Side (left vs. right)	0.5776	<0.001	0.4971	0.6711
Histopathology				
-Adenocarcinoma NOS	1 (reference)			
-Low differentiated	1.8784	<0.001	1.3637	2.5875
-Mucinous	1.4181	0.002	1.1377	1.7676
-Other	2.1560	0.006	1.2510	3.7158
dMMR ***	1.7209	<0.001	1.4689	2.0161
N-category—false negative rate				
Age	0.9942	0.194	0.9855	1.0030
Sex	1.0800	0.408	0.8998	1.2963
BMI	1.0069	0.459	0.9887	1.0254
Comorbidity (+/−)	1.1980	0.053	0.9973	1.4390
Comorbidity (cont)	1.0727	0.021	1.0106	1.1388
MDT meeting	1.2431	0.066	0.9856	1.5679
Side (left vs. right)	2.1859	<0.001	1.8088	2.6416
Histopathology				
-Adenocarcinoma NOS	1 (reference)			
-Low differentiated	0.4651	<0.001	0.3316	0.6523
-Mucinous	0.6493	0.005	0.4806	0.8773
-Other	0.7473	0.349	0.4061	1.3750
dMMR ***	0.5954	<0.001	0.4646	0.7630

* Odds ratio. ** Confidence interval. *** Mismatch repair deficient.

**Table 4:** Crosstabulations of false positive rates, false negative rates, false discovery rates and false omission rates against tumor sidedness and mismatch repair status.

Category	Diagnostic Test vs. Gold Standard		Rate of Faulty Preoperative Staging	
T3–4 vs. T0–2				
	False positive rate (=1-specificity)		False discovery rate (=1-PPV)	
	(Percent, with 95% CI in parentheses)		(Percent, with 95% CI in parentheses)	
Tumor location	pMMR *	dMMR **	pMMR	dMMR
Left-sided	21% (18–25%)	11% (3–27%)	12% (10–14%)	8% (2–19%)
	(*n* = 559)	(*n* = 35)	(*n* = 987)	(*n* = 52)
Right-sided	22% (18–26%)	25% (20–31%)	10% (8–12%)	9% (7–12%)
	(*n* = 441)	(*n* = 255)	(*n* = 1021)	(*n* = 695)
	False negative rate (=1-sensitivity)		False omission rate (=1-NPV)	
	(Percent, with 95% CI in parentheses)		(Percent, with 95% CI in parentheses)	
Tumor location	pMMR	dMMR	pMMR	dMMR
Left-sided	33% (30–35%)	23% (13–35%)	49% (46–52%)	31% (18–47%)
	(*n* = 1295)	(*n* = 62)	(*n* = 867)	(*n* = 45)
Right-sided	36% (33–38%)	26% (23–29%)	60% (56–63%)	54% (49–59%)
	(*n* = 1434)	(*n* = 853)	(*n* = 854)	(*n* = 413)
N+ vs. N0				
	False positive rate (=1-specificity)		False discovery rate (=1-PPV)	
	(Percent, with 95% CI in parentheses)		(Percent, with 95% CI in parentheses)	
Tumor location	pMMR	dMMR	pMMR	dMMR
Left-sided	27% (24–29%)	36% (26–47%)	52% (48–56%)	77% (61–89%)
	(*n* = 1216)	(*n* = 84)	(*n* = 627)	(*n* = 39)
Right-sided	35% (32–38%)	44% (41–48%)	46% (43–49%)	64% (60–68%)
	(*n* = 1142)	(*n* = 801)	(*n* = 871)	(*n* = 558)
	False negative rate (=1-sensitivity)		False omission rate (=1-NPV)	
	(Percent, with 95% CI in parentheses)		(Percent, with 95% CI in parentheses)	
Tumor location	pMMR	dMMR	pMMR	dMMR
Left-sided	57% (53–61%)	53% (29–76%)	31% (28–33%)	16% (8–27%)
	(*n* = 698)	(*n* = 19)	(*n* = 1287)	(*n* = 64)
Right-sided	39% (36–43%)	34% (29–40%)	29% (27–32%)	19% (16–22%)
	(*n* = 778)	(*n* = 306)	(*n* = 1049)	(*n* = 549)

* MMR deficient. ** MMR proficient.

## Data Availability

The data are publicly available in the Danish DCCG database.
